# HIV-2 Neutralization Sensitivity in Relation to Co-Receptor Entry Pathways and Env Motifs

**DOI:** 10.3390/ijms23094766

**Published:** 2022-04-26

**Authors:** Zsófia Ilona Szojka, Sara Karlson, Emil Johansson, Gülşen Özkaya Şahin, Marianne Jansson

**Affiliations:** 1 Department of Laboratory Medicine, Lund University, 221 84 Lund, Sweden; sara.karlson@med.lu.se (S.K.); emil.johansson@med.lu.se (E.J.); marianne.jansson@med.lu.se (M.J.); 2 Department of Translational Medicine, Lund University, 205 02 Malmö, Sweden; ruhidil_gulsen.ozkaya_sahin@med.lu.se; 3 Department of Clinical Microbiology, Laboratory Medicine, Skåne University Hospital, 222 42 Lund, Sweden

**Keywords:** HIV-2, neutralization sensitivity, target cell co-receptors, Env motifs

## Abstract

HIV-2, compared to HIV-1, elicits potent and broadly neutralizing antibodies, and uses a broad range of co-receptors. However, both sensitivity to neutralization and breadth of co-receptor use varies between HIV-2 isolates, and the molecular background is still not fully understood. Thus, in the current study, we have deciphered relationships between HIV-2 neutralization sensitivity, co-receptor use and viral envelope glycoprotein (Env) molecular motifs. A panel of primary HIV-2 isolates, with predefined use of co-receptors, was assessed for neutralization sensitivity using a set of HIV-2 Env-directed monoclonal antibodies and co-receptor indicator cell lines. Neutralization sensitivity of the isolates was analysed in relation target cell co-receptor expression, in addition to amino acid motifs and predicted structures of Env regions. Results showed that HIV-2 isolates were more resistant to neutralizing antibodies when entering target cells via the alternative co-receptor GPR15, as compared to CCR5. A similar pattern was noted for isolates using the alternative co-receptor CXCR6. Sensitivity to neutralizing antibodies appeared also to be linked to specific Env motifs in V1/V2 and C3 regions. Our findings suggest that HIV-2 sensitivity to neutralization depends both on which co-receptor is used for cell entry and on specific Env motifs. This study highlights the multifactorial mechanisms behind HIV-2 neutralization sensitivity.

## 1. Introduction

Human immunodeficiency viruses type 1 (HIV-1) and type 2 (HIV-2) are causative agents of the acquired immunodeficiency syndrome (AIDS) [1,2]. While HIV-1 is pandemic, HIV-2, with an estimated 1–2 million infected individuals, is mostly confined to West Africa and countries that share past socio-economic relation with this region [1]. HIV-2 is less transmissible and less pathogenic [1], and the median time to AIDS is approximately twice as long in untreated HIV-2 infected individuals compared to untreated HIV-1 infected individuals [3]. Moreover, compared to HIV-1 infected individuals, the plasma viral load is lower in HIV-2 infected individuals at the setpoint and in individuals matched for CD4+ T-cell count [4,5,6]. Although the precise mechanism behind lower plasma viral load in HIV-2 infection is still not fully understood, a broader and more potent immune response has been suggested to be a contributing factor [7,8,9,10,11,12,13,14,15]. Thus, previous studies have reported on potent and broad neutralizing antibodies distinguishing HIV-2 from HIV-1 infection [8,11,13,16]. Neutralization escape is also rare in HIV-2 infection, compared to HIV-1 infection [17,18]. Still, several studies have described HIV-2 strains with varying sensitivity to neutralizing antibodies [8,11,13]. 

As in the case of HIV-1, HIV-2 infection of a target cell begins with the interaction between the outer viral envelope glycoprotein (Env), gp125, and CD4, which initiates configurational changes in Env, which in turn triggers binding to the co-receptor [19]. However, one characterizing feature of HIV-2, compared to HIV-1, is the ability to use, beside CCR5 and CXCR4, a range of alternative co-receptors, including but not limited to CCR1, CCR2b, CCR3, CCR8, CXCR6, GPR15 [20,21]. Still, efficient infection of peripheral blood mononuclear cells (PBMC) by HIV-2 isolates have been attributed to the use of CCR5 or CXCR4 [22]. However, it cannot be excluded that alternative co-receptors may play role during infection of other target cells [23]. In line with that noted during HIV-1 infections, co-receptor tropism switch from primarily CCR5-use, to also inclusion of CXCR4-use, can be detected in HIV-2 infected individuals [18]. Alterations in Env V3 region amino acids composition, including elevated net charge, have been linked to CXCR4-use of both HIV-1 and HIV-2 [24,25]. The presence of positively charged amino acids at the 11th and the 25th amino acid in the V3 loop and net charge greater than +6 (for HIV-2) has consistently been associated with CXCR4-use [24,25]. Doring et al. also showed that valine insertion at position 25 of the HIV-2 V3 loop can be associated with CXCR4-use [26]. Primarily CCR5 tropic HIV-2 variants are transmitted, and it has been shown that most HIV-2-infected individuals produce potent C2V3C3-specific neutralizing antibodies early in the infection [27]. The emergence of CXCR4-using viruses has been reported to lead to resistance to antibody neutralization [27]. Although HIV-2 strains that can infect target cells via CCR5 are maintained throughout the asymptomatic stage, alternative co-receptors (e.g., GPR15 and CXCR6) are also efficiently used, which emphasize that alternative co-receptors may play a role in the pathogenesis, for example by mediating immune escape [21]. Thus, diverse co-receptor use, beyond CCR5 and CXCR4 [20,21], and diverse neutralization sensitivities [8,11,13,15] have been described for HIV-2. However, knowledge on the association between neutralization sensitivity and alternative co-receptor use, and the relation to Env amino acid motifs, is limited for HIV-2.

In the current study we have analysed a panel of HIV-2 primary isolates, with diverse alternative co-receptor use, for neutralization sensitivity to HIV-2 Env-directed monoclonal antibodies (mAbs) while entering target cells via specific co-receptors. Our results suggest that HIV-2 sensitivity to neutralization depends both on which co-receptor is used for target cell entry and specific motifs in the V1, V2 and C3 regions of the outer HIV-2 Env protein.

## 2. Results

### 2.1. Characterisation of HIV-2 Co-Receptor Use in Relation V3 Sequence Motifs

We initially set out to determine, verify, and relatively quantify, co-receptor use of a panel of ten HIV-2 primary isolates (Table 1). For this purpose we infected GHOST(3)-CD4 cells expressing different co-receptors, either CCR5, CXCR4, CXCR6 or GPR15, with the panel of HIV-2 isolates. Infection efficiency was determined by detection and quantification of plaque forming units (PFUs), i.e., GFP positive cells, by automated microscopy. All HIV-2 isolates were determined to use CCR5 for target cell entry. The second most commonly used co-receptor was the alternative co-receptor GPR15, which all analysed isolates, except 12524, used. Here the GPR15-use appeared to be efficient in seven out of the nine isolates. CXCR6-use was also common, and found to be used by seven isolates, although 1816 and 02va425 isolates used CXCR6 weakly (PFU **≤** 25). Instead, CXCR4 was only used by three of the isolates, 1010, 01va566 and 12524.

**Figure 1:**
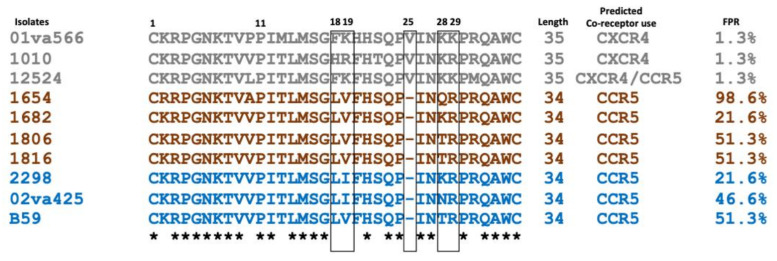
V3 loop amino acid sequence alignment and prediction of CXCR4-use by geno2pheno[coreceptor-hiv2] online server. Amino acid numbered according to the first cysteine (C) residue of the HIV-2 Env V3 loop region. The black boxes highlight the positions 18, 19, 25, 28 and 29 of the V3 loop, (*) indicate highly conserved residues and (-) represents a gap. The FPR (false positive rate) represents the probability of incorrect classifying a non-CXCR4-using (R5 virus) as a CXCR4-user, where the FPR cut off is 5% [26].

The V3 region of the viral Env surface glycoprotein has been reported to be the main determinant for CCR5 and/or CXCR4-use of both HIV-1 and HIV-2 [24,25,28]. Thus, in order to associate Env sequence motif with co-receptor use of the ten studied HIV-2 primary isolates, we next analysed the amino acid sequence of the Env V3 loop. Considering the phenotypically determined co-receptor use and the V3 sequences, the HIV-2 isolates could be classified into three groups (Table 1 and Figure 1); (i) isolates able to use CXCR4 in addition to other co-receptors, displaying a V3 loop with net charge equal to +7 including positively charged amino acids, such as arginine (R), and lysine (K) at the position 18, 19, 28 and 29 and a valine (V) insertion at position 25, in addition to predicted CXCR4-use (false positive rate 1.3%) according to geno2pheno[coreceptor-hiv2] [26]; (ii) isolates with efficient use of the alternative co-receptor GPR15, with L and V at V3 position 18 and 19, and a net charge lower or equal to +6; (iii) isolates with low or moderate capacity to use alternative co-receptor GPR15, with L and V/I at V3 position 18 and 19, and a net charge lower or equal to +6.

To determine if the panel of HIV-2 isolates analysed in the current study was representative of HIV-2 V3 sequences obtained from patient material, without virus isolation and propagation, and previously reported in relation to Env sequence motifs (i.e., V3 amino acids 18, 19, 25, 28 and 29), we analysed 82 V3 sequences downloaded from the Los Alamos National Laboratory HIV sequence database (https://www.hiv.lanl.gov, accessed on 18 February 2022). These sequences were derived from HIV-2-infected individuals classified as asymptomatic (*n* = 32), symptomatic (*n* = 29), or diagnosed with AIDS (*n* = 21) (Figure S1a–c, see Supplementary Materials). We found the V25 insertion in sequences from 3% asymptomatic, 17% symptomatic and 19% AIDS diagnosed individuals. Moreover, amino acid position 28 was found to be quite variable among the sequences, R28 was observed in all asymptomatic sequences, positively charged R and K amino acids were observed in 48% of symptomatic and in 45% of AIDS sequences. Moreover, V or isoleucine (I) were observed at position 19 in sequences of 97% asymptomatic, 83% symptomatic, and 81% of AIDS diagnosed individuals, and leucine (L)18 and R29 residues were highly conserved, irrespectively of disease status (Figure S2a,b). These results suggest that the V3 Env motifs linked to co-receptor use are also associated with disease progression.

### 2.2. HIV-2 Sensitivity to Neutralizing Antibodies Is Associated with Co-Receptor Entry-Pathway

**Figure 2:**
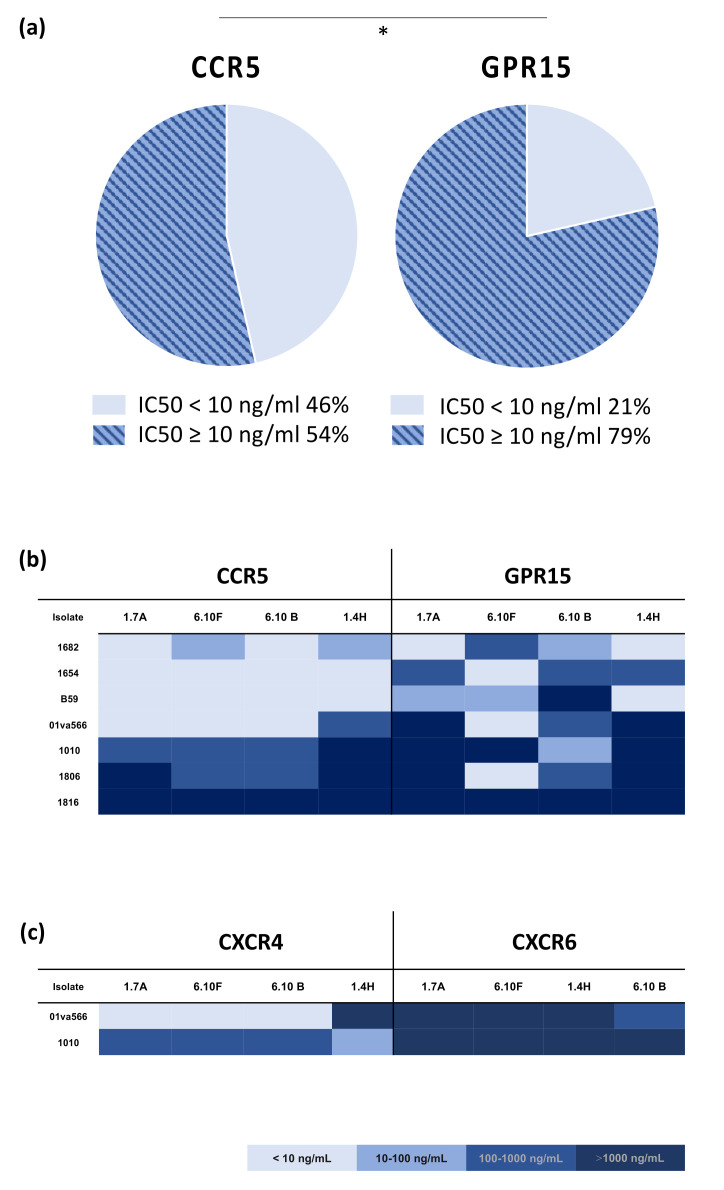
HIV-2 sensitivity to neutralizing by monoclonal antibodies when entering target cells through different co-receptors. (**a**) Pie-charts illustrating percentage of assays (*n* = 28) where the inhibitory concentration 50% (IC_50_) were <10 or ≥10 ng/mL, when the four different HIV-2 Env-directed monoclonals (mAbs) were tested against the panel of HIV-2 isolates (*n* = 7) using GHOST (3) cells either expressing CCR5 or GPR15. Chi-square analysis was used to compare proportion of HIV-2 neutralization assays with IC_50_ values under and above 10 ng/mL. * *p* < 0.05. IC_50_ spectra, <10, 10–100, 100–1000 or >1000 ng/mL of four mAbs when assayed against HIV-2 isolates infecting cells via (**b**) CCR5 or GPR15 and (**c**) CXCR6 or CXCR4.

Although it is well established that HIV-2 has the ability to enter target cells through the interaction with a range of alternative co-receptors, it is not clear whether HIV-2 co-receptor entry pathway impacts the sensitivity to neutralizing antibodies. Thus, we set out to evaluate the sensitivity of HIV-2 isolates entering cells through the interaction with different co-receptors. For this purpose, we analysed the sensitivity of the panel of HIV-2 primary isolates to neutralization by HIV-2 V3 region (1.7A and 6.10F), CD4 bindings site (CD4bs) (6.10B) and CD4-induced (CD4i) (1.4H) epitope specific mAbs [11] during infection of co-receptor indicator GHOST (3) cell lines expressing either CCR5, CXCR4, GPR15 or CXCR6,. Initially, we noted that the neutralization sensitivity of the ten HIV-2 isolates varied widely, from IC_50_ < 10 ng/mL to >1000 ng/mL, when assessed against the different mAbs using the CCR5-expressing cells (Figure S3). However, a large proportion of isolates (6 out of 10) were highly sensitive (IC_50_ < 10 ng/mL) against one or more of the of the analysed mAbs (Figure S3). When comparing neutralization sensitivity of the seven isolates that had the capacity to enter cells either via CCR5 or GPR15, we found that over all the virus neutralization sensitivity was reduced when GPR15, compared CCR5, was used for target cell entry (Figure 2a). Percentage of assays where the inhibitory concentration 50% (IC_50_) of the four mAbs was more than 10 ng/mL, was significantly higher when viruses entered target cells via GPR15, as compared to via CCR5, Chi-square, *p* < 0.05 (Figure 2a). 

When dissecting sensitivity of the isolates to the four antibodies we found that IC_50_ values ranged from <10 ng/mL to >1000 ng/mL in all four cases (Figure 2b). Thus, some isolates (1682, 1654, B59 and 01va566) were highly sensitive, especially when tested in cultures of the CCR5 expressing cells, whereas other isolates (1806, 1806 and 1010) were found to be highly resistant when analysed using either the CCR5 or GPR15-expressing cells. This resistance was especially prominent against neutralization by the antibodies directed against the V3-(1.7A) and the CD4i-epitopes (1.4H). Moreover, when comparing neutralization sensitivity of the two viruses able to use CXCR4 and CXCR6, we observed that none of the monoclonal antibodies showed strong inhibition of these isolates when CXCR6 was used as a co-receptor (Figure 2c). Instead, neutralization sensitivity of the two viruses using CXCR4 entry pathway was divergent, one sensitive and one resistant (Figure 2b,c). 

These results, taken together, indicate that neutralization sensitivity of HIV-2 isolates is influenced by co-receptor entry pathway. Thus, infection mediated by alternative co-receptor use appears to be more resistant to neutralization by monoclonal antibodies than entry via CCR5. 

### 2.3. Alterations in the HIV-2 Env V1/V2 Regions in Relation to Neutralization Sensitivity 

**Figure 3:**
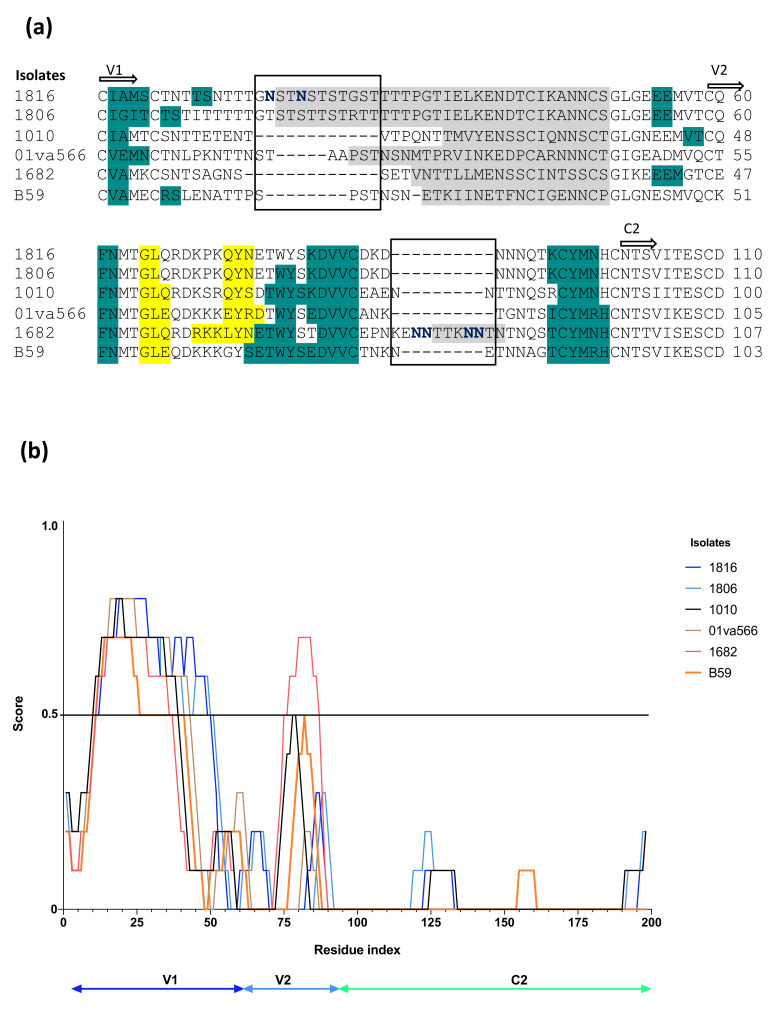
Characterization of the Env V1/V2 regions of HIV-2 isolates with CCR5 and GPR15 use. (**a**) Secondary structure prediction of V1/V2 regions of HIV-2. Teal dark green represents beta strand, yellow represents alpha-helix, while the grey represents the predicted disordered regions. Black boxes indicate regions with insertions. Potential N-linked glycosylation sites within the insertions are represented in dark blue. (**b**) Disorder prediction of the V1/V2 and C2 regions of HIV-2 Env. Prediction was performed using IUPred3 and SPOT-Disorder2 online servers [31,32]. Cut-off is represented with line at score 0.5.

After analysing HIV-2 neutralization sensitivity in relation to co-receptor use, we set out to study its relationship to amino acid motifs in the V1/V2 Env region, which has been reported to be a target for neutralizing antibodies, neutralization escape and also influence co-receptor use [21,30]. The V1/V2 region of six HIV-2 isolates able to use both CCR5 and GPR15 were obtained, and secondary structure predictions were performed based on Env sequences and homology model structures. The V1/V2 region sequence showed high degree of variability, which contrasts with the results for the V3 region, especially at the tip of the V1 region (Figure 3a). Notably, we found that isolates resistant to neutralization, both using CCR5 and GPR15, had a 12 amino acid long insertion in the V1 region (1806: GTSTSTTSTRTT and 1816: GNSTNSTSTGST). Furthermore, disorder predictions suggested that this part of the V1 region was unstructured in all of the analysed isolates (Figure 3a,b and Figure S4a,b). However, the disordered region was especially elongated in the most resistant isolates, 1806 and 1816 (Figure 3a,b). In the case of the most resistant isolate 1816, we also noted two additional potential N-linked glycosylation sites in the insertion after the V1 tip region (Figure 3a). Instead, 1682 which was the overall most neutralization sensitive isolate in our study, had a 10 amino acid long insertion, including four potential N-linked glycosylation sites, in the V2 region (Figure 3a). 

To investigate if the studied panel of HIV-2 isolates was representative of previously characterized HIV-2 strains, we analysed the length of insertions in either V1 or V2 regions from 48 (V1) and 50 (V2) HIV-2 sequences downloaded from the Los Alamos National Laboratory HIV sequence database (http://www.hiv.lanl.gov, accessed on 18 February 2022) (Table S1). The length variability of the V1 region was analysed in sequences from individuals with various disease stages, asymptomatic (*n* = 15), symptomatic (*n* = 11) and with AIDS (*n* = 12) (Figure S5a). Length of the insertion in the V1 region was significantly longer in individuals with AIDS as compared to those with asymptomatic infection according to the non-parametric Mann-Whitney analysis (*p* = 0.0162) (Figure S6a). Next length variation in the V2 region was analysed among individuals assessed to be asymptomatic (*n* = 17), symptomatic (n = 11) and with AIDS (*n* = 12) (Figure S5b). Here, no significant variation in length of V2 was noted between the different groups (Figure S6b). 

Thus, these findings taken together, may suggest that insertion in the V1 region, associated with neutralization resistance in our study, can be detected during asymptomatic HIV-2 infection, but appears to be enlarged during disease progression.

### 2.4. Alterations in the HIV-2 Env C3 Region in Relation to Neutralization Sensitivity 

**Figure 4:**
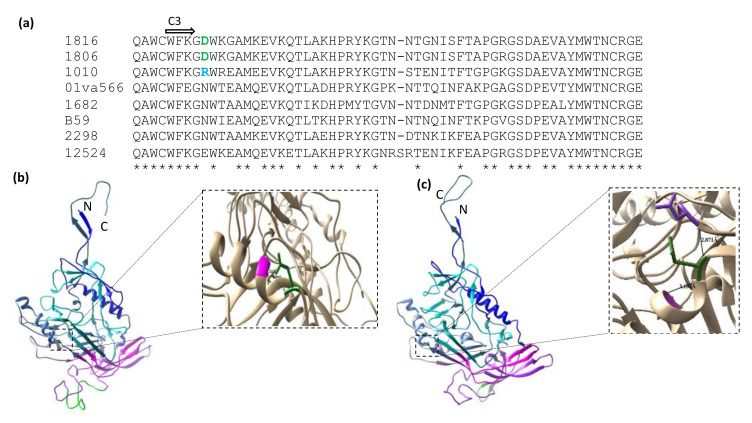
Effect of amino acid polymorphisms in the A2 helix of the gp125 C3 region. (**a**) Amino acid alignment of N-terminal region of C3 domain of HIV-2 isolates. (**b**) Homology model of 1816 Env. (**c**) Homology model of 1682 Env. The 3-dimensional gp120 monomers were obtained from Swissmodel repository. The different domains represented by different colours: C1 is blue; V1/V2 purple, whereas the insertion in this region is green; C2 is cyan blue; V3 is magenta; C3 is cornflower blue; V4 is plum; C4 is dark cyan; V5 is salmon; C5 is steel blue. Critical amino acids in the 5th position of the C3 region is represented with forest green and formed hydrogen bonds are represented with black lines for both of the isolates. “-” indicates lack of insertion in sequences except, isolate 12524. Consensus across tested HIV-2 isolates’ sequences aligned is indicated below each amino acid residue by symbol “*”. The model structures of gp125 were obtained from the Swissmodel repository.

We next explored potential links between neutralization sensitivity and the structure of the HIV-2 Env C3 region. A previous study reported that even a single substitution in alpha2 helix of the C3 region could lead to escape from neutralizing antibodies in HIV-1 [33]. Here, we observed that the most resistant isolates, 1816 and 1806, had an aspartic acid at position 5 (D5) within the C3 region, and the 1010 and 12524 isolates, which also displayed a neutralization resistant phenotype, had charged amino acids, R and glutamic acid (E), in the same position. Instead, the more neutralization sensitive isolates had an asparagine (N) at this position, which also predicted a potential N-linked glycosylation site (Figure 4a). Furthermore, structural modelling suggested that the D5 residue in the C3 domain of the resistant isolates formed two H-bonds with the 8th glycine (G) (2.737 Å) and 9th alanine (A) (3.066 Å) residues in the alpha2 helix. Instead, the D5 position in the 1682 isolate, being the most neutralization sensitive isolate, formed an H-bond with A9 (3.104 Å) in the C3 domain, and the N95 (2.871 Å) C2 region (Figure 4c). Thus, structural alterations within the alpha2 helix of the HIV-2 Env C3 region, similar to that reported for HIV-1 [33], appeared to alter HIV-2 neutralization sensitivity.

## 3. Discussion

This study reveals that HIV-2 sensitivity to neutralizing antibodies is affected by co-receptor entry pathway. Notably, HIV-2 resistance to neutralization appears to be more common at the use of the alternative co-receptor GPR15, as compared to the use of CCR5, during target cell entry. Our data also suggest that neutralization sensitivity of HIV-2 can be altered by different V1/V2 and C3 Env motifs.

It is well established that individuals with HIV-2, compared to HIV-1 infection, display a broad and potent neutralizing antibody response [8,13,15]. HIV-2, compared to HIV-1, may also use a broader range of alternative co-receptors for target cell entry [20]. However, to the best of our knowledge, this study is the first to report on the relation between alternative co-receptor entry pathway and neutralization sensitivity of HIV-2.

Our neutralization studies revealed that most of the isolates were sensitive to neutralization when CCR5 was used for viral entry, while the use of GPR15 resulted in reduced neutralization sensitivity. Previously, it has been shown that HIV-2 neutralization sensitivity is reduced when the virus has the ability to use CXCR4 [27]. These findings taken together suggest that neutralization sensitivity can be influenced by the occlusion or exposure of certain Env epitopes due to differences in conformational changes following interaction with different co-receptors. It is known that GPR15, similarly to CCR5, contains tyrosines in the N-terminus, however, it lacks the third extracellular loop [34,35], which may impose structural alterations following HIV-2 Env binding.

Alternative co-receptor use, such GPR15 and CXCR6, can be observed in both asymptomatic and symptomatic HIV-2-infected individuals, and both of these co-receptors are also commonly used by different simian immunodeficiency virus (SIV) strains [34,36,37,38]. In contrast, GPR15 use by HIV-1 isolates is more rare, but has been documented [39]. Furthermore, SIVmac239 have been shown to use GPR15 for viral entry with similar efficiency as CCR5 [40]. This is in line with our observation that most of the isolates could use both CCR5 and GPR15, for entry of cells expressing respective co-receptor. Intriguingly, the 1682 isolate, the most neutralization sensitive isolate in the tested panel, has previously been described to have the capacity to enter cells in a CD4-independent manner [41]. This feature, which some HIV-2 strains display, has also been linked to neutralization sensitivity [13].

GPR15 is expressed in colonic mucosa, lymph node, prostate, testis, bladder and liver [35,42], which may implicate its importance in the replication of virus in these types of tissues. Especially the gut-associated lymphoid tissue (GALT) has been shown to contain a large reservoir of target cells for HIV, and has been shown to be an important compartment containing potentially replication-competent HIV-1 DNA in ART-receiving individuals [43,44]. Expression of GPR15 in the basal surface of the epithelium was found to coincidence with significantly increased SIV virion binding in the gut, which may induce HIV enteropathy [45]. In aviremic HIV-2-infected individuals, it has been reported that the virus can replicate in GALT, even though mucosal CD4 T-cell depletion was not evident, and that this was accompanied by homing of FOXP3-positive regulatory T-cells (T-regs) [35,46,47]. Interestingly, in a recently published study, a new effective HIV inhibitory pathway was discovered [48]. Cystatin C was able to prevent GPR15-dependent HIV and SIV infection without interfering the binding of the agonistic C-C chemokine ligand (GPR15L) [48]. Thus, although HIV-2 entering cells via GPR15 seems to be more resistant to neutralizing antibodies, other virus inhibitory mechanisms may be at play in GALT. An alternative explanation of lower depletion of CD4+ cells in the GALT of HIV-2 infected, compared to HIV-1 infected individuals, might also be explained by the suggested intracellular neutralizing effect that has been reported for secretory IgAs [49]. Further studies are required to determine this possibility.

To further understand traits that impacts HIV-2 neutralization sensitivity we set out to characterize different Env regions previously linked to neutralization sensitivity. While the V3 loop of HIV-1 Env plays a crucial role as an immunodominant determinant for neutralizing antibodies [50], the HIV-2 V3 region is not as immunodominant [51]. Previous in silico predictions have suggested that substitutions in the HIV-2 V3 loop might lead to the loss of H-bond, which influence the surface exposition of the V3 loop [52]. However, in the current study we could not find any links between V3 region amino acid motifs and neutralization sensitivity. Beside the V3 region, other variable regions, for example V1/V2, have also been reported to be targets for neutralizing antibodies, neutralization escape and also influence co-receptor use [21,30]. It was previously shown that mutations in the tip and base of V2; and in the base of the V1 region, influence the interaction between Env and co-receptors [21]. Moreover, mutations and glycosylations in the V1 region can affect the virus-induced syncytium formation [53]. Our sequence analysis revealed that neutralization sensitivity might be related to a highly disordered insertion in the V1 region. We also found that when this insertion contains potential N-linked glycosylation sites, it can lead to more resistance against neutralization. This is supported by previous findings revealing that potential N-linked glycosylation sites in the HIV-1 V1/V2 region can influence the structure, which in turn may modulate the binding to cellular receptors [2]. We also noted a ten amino acid insertion in the V2 region of the most sensitive isolate 1682, which is in line with the finding that the V2 loop also can affect the sensitivity to neutralization [54]. 

Beside Env variable regions, the C3 region also contains major antigenic targets and it is important for receptor binding [55]. Both the C2 and C3 regions of HIV-2 include positively selected sites, which has been associated with high solvent exposure [55,56]. This may lead to better antibody accessibility, which in turn might play role in neutralization sensitivity. In addition, amino acid substitutions in the alpha2 helix in the C3 domain of HIV-1 Env can directly mediate neutralization escape [33]. Notably, our sequence analysis revealed that the most resistant HIV-2 isolates contain aspartic acids at the 5th residue (N5) of the C3 domain, while the sensitive isolates contain aspartate at the same position. It was also predicted that the N5 residue formed two H-bonds with other residues in the C3 domain. In agreement, a previous study reported that HIV-1 neutralization escape was associated with alteration of the alpha2 helix in C3 [33].

In conclusion our results suggest that HIV-2 isolates are more resistant to neutralizing antibodies when entering target cells via the alternative co-receptors. Sensitivity to neutralizing antibodies appears also to be linked to specific HIV-2 Env motifs. Taken together, we believe our study adds to the understanding of the multifactorial mechanisms that contributes to the variation of HIV-2 neutralization sensitivity, and also provides novel insights into the impact that different virus co-receptor entry pathways may have on HIV-2 neutralization. Thus, our findings should spur further studies on the impact of virus-host interactions for appreciation of HIV neutralization sensitivity at the entry of different target cells.

## 4. Materials and Methods

### 4.1. Panel of HIV-2 Primary Isolates

For the analysis of neutralization sensitivity and sequencing of *env*, ten subtype A HIV-2 primary isolates were used (Table 1). All isolates (1010, 01va566, 12524, 1654, 1682, 1806, 1816, 2298, 02va425, B59) originated from individuals either living, or moving out from West Africa [18,20,22,29].

Virus stocks were prepared by propagation in stimulated human primary CD4+ T cells. In brief, PBMCs of healthy blood donors were stimulated with phytohemagglutinin (Sigma-Aldrich, St. Louis, MO, USA) for three days, as previously described [57]. CD4+ T cells were the enriched using MagniSort Human CD4 Memory T cell Enrichment Kit (Thermo Fisher Scientific, Waltham, MA, USA), according to the manufacturer’s instructions. Virus propagation was done, as described [57] in RPMI-1640 medium (Gibco, Paisley, UK) supplemented with 10% FBS (Sigma- Aldrich, St. Louis, MO, USA), penicillin-streptomycin (Sigma-Aldrich, St. Louis, MO, USA), 10 units/mL interleukin-2 (Amersham Pharmacia Biotech, Uppsala, Sweden) and 2 μg/mL polybrene (Sigma, Neustadt an der Weinstraße, Germany). Freshly stimulated donor CD4+ T-cells were added once a week to the virus cultures and cell free supernatants were harvested 7, 14 and 21 days after infection, aliquoted and stored at −80 °C until use.

### 4.2. PCR Amplification and Sequencing of HIV-2 Env Regions

HIV-2 viral RNA was extracted from 200 μL of HIV-2 culture supernatant in the presence of carrier RNA (Qiagen, Hilden, Germany), using Qiagen miRNeasy micro kit (Qiagen, Hilden, Germany) following the manufacturer’s protocol. cDNA synthesis was performed on extracted RNA using SuperScript III One-Step RT-PCR System (Thermo Fisher Scientific, Waltham, MA, USA), R1 primer (5′-GGT CAT CAT CAT Cwr mAT CTA yAT C-3′), and RiboLock RNase inhibitor (Invitrogen, Taastrup, Denmark) at an initial 60 min incubation at 50 °C, followed by another 60 min at 55 °C, and a final heat-inactivated step at 85 °C for 5 min. The cDNA was diluted to the concentration where 33% or less of the PCR reactions were positive. 

Amplification of the full-length *env* was performed using the DreamTaq™ DNA Polymerase (Thermo Fisher Scientific, Waltham, MA, USA) in a nested first PCR setup, where the first PCR was done using forward and reverse primers F31 and R2, and the nested PCR was done using forward and reverse primers F42 and R3. Both first and nested-PCRs were carried out with an initial denaturation for 2 min at 95 °C, followed by 40 cycles of 2 min at 95 °C, 30 s at 55 °C, 3 min at 72 °C, and a final elongation step for 10 min at 72 °C.

The V1-C3 region of HIV-2 *env* gene was amplified using a previously described protocol [56], with minor modification. In brief, DreamTaq™ DNA polymerase was used and the forward and reverse primers KH2-OF and TH2-OR were used for the outer PCR, and the forward and reverse primers KH2-OF and KH2-OR were used for the nested PCR.

At sequencing of the V1-C3 region, and also the full-length HIV-2 *env,* nested PCR primers and sequencing primers, respectively, were used, as indicated in Table S2. Sanger sequence reactions were performed by BigDye™ Terminator v1.1 Cycle Sequencing (Life Technologies, Carlsbad, CA, USA), as per the instructions of the manufacturer, and the generated sequences were analyzed on an automated DNA Sequencing instrument (Applied Biosystems, Inc., Norwalk, CT, USA) at the Lund University DNA sequencing facility. HIV-2 full-length and V1-C3 region *env* sequences were edited using CodonCode Aligner v1.5.2 and MEGA5 using the Clustal algorithm [58]. 

### 4.3. Analysis of Globally Accessible HIV-2 Env Sequences 

HIV-2 V1/V2 and V3 regions’ sequences from infected individuals were downloaded from the HIV sequence database (https://www.hiv.lanl.gov, accessed on 18 February 2022) with the purpose to determine the sequence diversity of the variable regions among different HIV-2 groups in relation to amino acid sequence variance of different HIV-2 isolates. The amino acid variability of sequences was schematically visualized using Weblogo (for the V1-V2 region and for C2V3C3 region) (https://weblogo.berkeley.edu/logo.cgi, accessed on 18 February 2022).

### 4.4. Modelling and In Silico Predictions of HIV-2 Env Motifs and Structures

For in silico predictions, a molecular model of monomeric HIV-2 Gp125 structures were obtained from Swissmodel repository (SWISS-MODEL Template Library identifiers, PDB ID 5cay and 2B4C). Figures were created with the Chimera (https://www.cgl.ucsf.edu/chimera, accessed on 21 February 2022) and Pymol (DeLano Scientific LLC, Palo Alto, Santa Clara, CA, USA, accessed on 21 February 2022) softwares. A multiple-sequence alignment of amino acid sequences (without any gap) was made using ClustalW (https://www.genome.jp/tools-bin/clustalw, accessed on 22 February 2022). Potential N-glycosylation site prediction was performed using the N-glycoSite prediction tool from the HIV sequence database (https://www.hiv.lanl.gov/content/sequence/GLYCOSITE/glycosite.html, accessed on 23 February 2022) [59]. For short disordered predictions, the IUPred3 webserver (https://iupred3.elte.hu, accessed on 23 February 2022) [31] and SPOT-Disorder2 online server was used (https://sparks-lab.org/server/spot-disorder2/ accessed on 24 February 2022) [32]. Secondary structure prediction was determined in PSIPRED server (http://bioinf.cs.ucl.ac.uk/psipred, accessed on 25 February 2022) [60]. 

### 4.5. Production and Purification of Monoclonal Antibodies

Plasmids encoding four HIV-2 Env-specific neutralizing monoclonal antibodies (mAbs), 1.7A (recognizing V4 region and base of V3 loop); 6.10F (V3 region); 6.10B (CD4 binding site); 1.4H (unknown site of CD4 binding site) [11] was kindly provided by Prof James Robinson, Tulane University. HEK293T human embryonic kidney cells (Invitrogen, CA, USA) were transfected with light and heavy chain encoding plasmids at a 1:1 ratio, as previously described [61]. In brief, HEK293T cells were cultured in Dulbecco’s Modified Eagle’s Medium (DMEM) (Sigma-Aldrich, St. Louis, MO, USA), supplemented with 10% fetal bovine serum (FBS), glutamine and penicillin-streptomycin, and transfected with plasmid DNA using polyethylenimine (Sigma-Aldrich, St. Louis, MO, USA). Medium, that had been replaced 6hrs after transfection, was collected 48 h after transfection and filtered through a 0.45 µm polyvinylidene fluoride filter (Merck Millipore, Darmstadt, Germany). IgG from medium supernatants was purified using Protein G GraviTrap columns (Sigma-Aldrich, St. Louis, MO, USA), according to the manufacturer’s protocol. Collected elution fractions were desalted using PD-10 Desalting Columns (Cytivia, Uppsala, Sweden) and fractions were eluted in sterile water. The collected fractions were then concentrated using 30 K Amicon ultra centrifugation filters (Sigma-Aldrich, St. Louis, MO, USA) (4500 RPM, 20 min, 4 °C), and mAbs were portioned, and stored at 4 °C. IgG concentrations were analysed by ELISA [17,18], using AffiniPure goat anti-human IgG (20 µg/mL) (Jackson Immunotech, Cambridgeshire, UK) capture antibody and horseradish peroxidase (HRP) enzyme conjugated anti-human IgG (Thermo Fisher Scientific, Waltham, MA, USA) as detection antibody. ChromPure Human IgG (Jackson Immunotech, Cambridgeshire, UK) was used as a standard.

### 4.6. HIV-2 Neutralization Assay Using Co-Receptor Indicator Cell Lines 

GHOST (3) HIV co-receptor indicator cell lines derived from a clone of human osteosarcoma cells stably expressing CD4 together with one of the following chemokine receptors, CCR5, CXCR4, GPR15 or CXCR6 [20], were used as target cells in the neutralization assays. 

GHOST (3) cells were cultured in 96-well plates in DMEM supplemented with 7.5% FBS, glutamine and penicillin-streptomycin at 37 °C and 5% CO_2_, as previously described [62]. Initially, HIV-2 isolate stocks were titrated, and co-receptor use was determined by incubating viral stocks, serially diluted in 5-fold steps, with GHOST (3) cultures for three days. Since GHOST (3) cells contain a *tat*-dependent HIV-2 LTR-GFP construct, successful HIV infection was detected by analysis of green-fluorescent protein (GFP) using an automated microscopy and image-based method previously described [62]. Neutralization assay was setup by incubating HIV-2 supernatants (resulting in 30–80 PFUs as determined during titration for each separate cell line) with the separate mAbs at the following final concentrations: 1000, 100, 10 and 1 ng/mL. In parallel virus was incubated with medium. The virus and antibody or medium mixtures were incubated for one hour, then added to the cells, followed by further incubation at 37 °C for three days, including medium change after one day. At day three, PFU, i.e., GFP positive cells, were enumerated using the aforementioned protocol and % neutralization was calculated based on the percentage of the PFU reduction in cultures containing mABs, compare to virus cultures without antibodies, by using the following formula: % neutralization = [(average of PFU with samples mAB)/(average of PFU with samples mAB)] × 100.

### 4.7. Statistical Analysis

Comparison of HIV-2 neutralization sensitivity in assays using different target cells was analysed using Chi-square analysis, whereas comparison of length of V1 and V2 variable regions of different HIV-2 subgroups was analysed with non-parametric Mann-Whitney and Kruskal-Walls test using GraphPad Prism 8.0.

## Figures and Tables

**Table 1:** Co-receptor use ^a^ and outer Env V3 loop amino acid motifs ^b^ of HIV-2 isolates.

Isolate ^c^	Disease State	CCR5	CXCR4	GPR15	CXCR6	18	19	Insertion 25	28	29	Charge
01va566	AIDS	+++++	+++++	+++++	+++++	F	K	V	K	K	+7
1010	AIDS	+++++	+++++	+++++	+++++	H	R	V	K	R	+7
12524	AIDS	+++++	++++	−−−−	−−−−	F	K	V	K	K	+6
1654	AIDS	+++++	−−−−	+++++	+++	L	V	−−−−	Q	R	+5
1682	AS	++	−−−−	++++	++	L	V	−−−−	K	R	+6
1806	AS	+++++	−−−−	+++++	+++++	L	V	−−−−	T	R	+5
1816	AS	+++++	−−−−	+++++	+/−−−−	L	V	−−−−	T	R	+5
2298	AS	++++	−−−−	+	−−−−	L	I	−−−−	K	R	+6
02va425	AIDS	+++	−−−−	++	+/−−−−	L	I	−−−−	N	R	+5
B59	AS	+++++	−−−−	+++	−−−−	L	V	−−−−	T	R	+5

^a^ Infectivity of primary HIV-2 isolates using GHOST(3)-CD4 cells expressing different co-receptors was represented as PFU. +++++: PFU over 200, ++++: PFU over 100, +++: PFU over 50, ++: PFU over 25, +: PFU less than 25, −−−−: No use of the tested co-receptor. ^b^ Amino acid residues at the positions of 18, 19, 25, 28 and 29 are represented in one letter form, F: phenylalanine; H: histidine; L: leucine; R: arginine; K: lysine; V: valine; I: isoleucine, and numbering is according to the first cysteine (C) of the V3 loop. The global net charge of V3 is shown on the right. ^c^ HIV-2 primary subtype A isolates originated from individuals living in or moving out from countries in West Africa, as previously described [18,20,22,29]. AS: Asymptomatic disease state.

## Data Availability

The data presented in this study are available on request from the corresponding author.

## Supplementary Materials

The following supporting information can be downloaded at: https://www.mdpi.com/article/10.3390/ijms23094766/s1.
