# Supplementary material for: HIV-2 Neutralization Sensitivity in Relation to Co-Receptor Entry Pathways and Env Motifs
Source: Int J Mol Sci. 2022 Apr 26;23(9):4766. doi: 10.3390/ijms23094766 (PMC9101540; doi:10.3390/ijms23094766)
Supplement: Supplementary file 1 [file ijms-23-04766-s001.zip › ijms-1670395-supplementary.pdf]

Supplementary Materials

(a)

Name of sequences

```
                                        1         11     1819     25  2829       Length
A.GM.-.MCN13.AY509259                   CKRPGNKTVVPITLMSGLRFHSQPVINKRPRQAWC 35
A.IN.-.NIM_2.DQ478590                   CKRPGSKTVVPITLMSGLVFHSQPI-SQRPRQAWC 34
A.IN.-.NIM2_a.EU574629                  CKRPGNKTVVPITLMSGLVFHSQPI-SQRPRQAWC 34
A.PT.2000.00PTHDECT_20.GU983917         CRRPGNKTVVPITLMSGLIFRSQPI-NKRPGQAWC 34
A.IN.-.NIM_5_a.EU599050                 CKRPGNKTVAPVTLMSGLIFHSQPI-NTRPRQAWC 34
A.IN.-.NIM_5.DQ478593                   CKRPGNKTVAPVTLMSGLIFHSQPI-NTRPRQAWC 34
A.IN.1999.NARI_H2_6_3.DQ870445          CRRPGNKTVVPITLMSGLVFHSQPI-NKRPKQAWC 34
A.PT.1997.MS.GQ331136                   CRRPGNKTVVPITLMSGLVFHSQPI-NKRPRQAWC 34
A.IN.2000.NARI_H2_15_3.DQ870472         CKRPGNKTVVPITLMSGLIFHSQPI-NNRPKQAWC 34
A.IN.2000.NARI_H2_8_3.DQ870451          CKRPGNKTVVPMTLMSGLIFHSQPI-NNRPRQAWC 34
A.IN.1999.NARI_H2_2_4.DQ870433          CKRPGNKTVVAITLMSGLVFHSQPI-NARPRQAWC 34
A.IN.-.NIM_7_a.EU639420                 CRRPGNKTVVPITLMSGLIFHSQPI-NQRPRQAWC 34
A.IN.-.NIM_7.DQ973526                   CRRPGNKTVVPITLMSGLIFHSQPI-NQRPRQAWC 34
A.GW.1994.A245.AF023879                 CKRPGNKTVVPITLMSGLIFHSQPI-NRRPRQAWC 34
A.GW.1989.89GW2298.L76427               CKRPGNKTVVPITLMSGLIFHSQPI-NKRPRQAWC 34
A.CI.-.patient_31307.U67371             CKRPGNKTVVPITLMSGLVFHSQPI-NNRPRQAWC 34
A.GM.1990.CBL24.U05353                  CKRPGNKTVVPITLMSGLVFHSQPI-NNRPRQAWC 34
A.IN.-.NIM_3.EU639424                   CKRPGNKTVVPITLMSGLVFHSQPI-NNRPRQAWC 34
A.CI.-.patient_41863.U67376             CKRPGNKTVVPITLMSGLVFHSQPI-NRRPRQAWC 34
A.PT.1997.ARM.AJ001163                  CKRPGNKTVVPITLMSGLVFHSQPI-NRRPRQAWC 34
A.PT.1997.ARM.L76739                    CKRPGNKTVVPITLMSGLVFHSQPI-NRRPRQAWC 34
A.SN.1986.ST_JSP4_27.M31113             CKRPGNKTVVPITLMSGLVFHSQPI-NRRPRQAWC 34
A.CI.-.patient_2000593.U67379           CKRPGNKTVVPITLMSGLVFHSQPI-NKRPRQAWC 34
A.GW.1989.89GW1682.L76420               CKRPGNKTVVPITLMSGLVFHSQPI-NKRPRQAWC 34
A.IN.1995.NARI_H2_5_4.DQ870442          CKRPGNKTVVPITLMSGLVFHSQPI-NKRPRQAWC 34
A.IN.1999.NARI_H2_7_2.DQ870448          CKRPGNKTVVPITLMSGLVFHSQPI-NKRPRQAWC 34
A.IN.2001.NARI_H2_1_4.DQ870430          CKRPGNKTVVPITLMSGLVFHSQPI-NKRPRQAWC 34
A.CI.-.patient_3502964.U67385           CKRPGNKTVVPITLMSGLVFHSQPI-NTRPRQAWC 34
A.IN.-.NIM_4_b.EU587007                 CKRPGNKTVVPITLMSGLVFHSQPI-NTRPRQAWC 34
A.IN.2000.NARI_H2_16_4.DQ870475         CKRPGNKTVVPITLMSGLVFHSQPI-NTRPRQAWC 34
A.IN.1996.NARI_H2_4_3.DQ870439          CKRPGNKTVVPITLMSGLVFHSQPI-NERPRQAWC 34
A.IN.2001.NARI_H2_3_3.DQ870436          CKRPGNKTVVPITLMSGLVFHSQPI-NERPRQAWC 34
                                        *********   ****** * ****.  ** ****
```

(b)

Name of sequences

```
                                    1         11     18 19    25  28 29      Length
A.IN.-.NIM_8.DQ973521               CKRPGNKTVVPITLMSGLIFHSQP-INNRPRQAWC 34
A.IN.1999.NARI_H2_9_3.DQ870454      CKRPGNKTVVPITLMSGLIFHSQP-INTRPRQAWC 34
A.PT.-.ALI.AF082339                 CKRPGNKTVVPITLMSGLIFHSQP-INKRPRQAWC 34
A.IN.1999.NARI_H2_12_3.DQ870463     CRRPGNKTVVPITLMSGLVFHSQP-INDRPRQAWC 34
A.PT.1997.MLC.GQ331135              CKRPGNKTVAPITLMSGLVFHSQP-INNRPRQAWC 34
A.IN.1999.NARI_H2_17_2.DQ870478     CKRPGNKTVLPITLMSGLVFHSQP-INTRPRQAWC 34
A.GW.1989.89GW1817.L76425           CKRPGNKTVAPITLMSGLVFHSQP-INTRPRQAWC 34
A.GW.1989.89GW336.L76429            CKRPGNKTVVPVTLMSGLVFHSQP-INKRPRQAWC 34
A.ES.1997.SS2.AF176768              CKRPGNKTVVPITLMSGLVFHSQP-INQRPRQAWC 34
A.ES.1997.SS3.AF176769              CKRPGNKTVVPITLMSGLVFHSQP-INKRPRQAWC 34
A.ES.1997.SS4.AF176770              CKRPGNKTVVPITLMSGLVFHSQP-INKRPRQAWC 34
A.SN.1996.M6960625CB.EU530903       CKRPGNKTVVPITLMSGLVFHSQP-INKRPRQAWC 34
A.SN.1996.M6960625CD.EU530905       CKRPGNKTVVPITLMSGLVFHSQP-INKRPRQAWC 34
A.SN.1997.M6970408CA.EU530913       CKRPGNKTVVPITLMSGLVFHSQP-INKRPRQAWC 34
A.SN1998MIN652ENVVLP3605EU530819    CKRPGNKTVVPITLMSGLVFHSQP-INKRPRQAWC 34
H2_01_AB.CI.1990.7312a.FB293476     CKRPGNKTVVPITLMSGLVFHSQP-INKRPRQAWC 34
H2_01_AB.CI.1990.7312A.L36874       CKRPGNKTVVPITLMSGLVFHSQP-INKRPRQAWC 34
A.GW.1989.89GW1808.L76421           CKRPGNKTVVPITLMSGLVFHSQP-INTRPRQAWC 34
A.IN.-.NIM_6.DQ478594               CKRPGNKTVVPITLMSGLVFHSQP-INTRPRQAWC 34
ASN1999MIN1974ENVPC3603.EU530878    CKRPGNKTVVPITLMSGLVFHSQP-INTRPRQAWC 34
ASN1999.MIN3473ENVPC3675EU530851    CKRPGNKTVVPITLMSGLVFHSQP-INTRPRQAWC 34
A.IN.-.NIM_1_a.EU639409             CKRPGNKTVVPITLMSGLVFHSQP-INRRPRQAWC 34
A.SN.1995.M6950315C1.EU530891       CKRPGNKTVVPITLMSGLVFHSQP-INRRPRQAWC 34
A.SN.1998.M6980428CA.EU530923       CKRPGNKTVVPITLMSGLVFHSQP-INRRPRQAWC 34
A.ES.1997.SS1.AF176767              CKRPGNKTVVPITLMSGFKFHSRPVINKRPRQAWC 35
A.PT.1997.ETP.GQ331132              CKRPGNKTVKPITLMSGYKFHSRPVINEKPRQAWC 35
A.PT.2000.00PTSJP3A.AY500199        SRRPGNKTVVPITLMSGMRFHSRPVINDKPKQAWC 35
A.PT.2003.03PTHDECT_47.GU983933     CKRPGNKTSVPITLMSGYRFHSQAVINTKPKQAWC 35
A.PT.2003.03PTHDECT_50.GU983934     CKRPGNKTVVPITLMSGYRFHSQAVINTKPKQAWC 35
                                    ********* *******  ****  **  * ****
```

(c)

Name of sequences

```
                                                                      Length
                               1         11      18 19     25  28 29
A.IN.2000.NARI_H2_13_1.DQ870467    CKRPGNRTVVPITLMSGLVFHSQP-INTRPKQAWC  34
A.IN.2001.NARI_H2_11_2.DQ870462    CKRPGNRTVVPITLMSGLVFHSQP-INTRPKQAWC  34
A.CI.1989.89CI1654.L76419          CRRPGNKTVAPITLMSGLVFHSQP-INQRPRQAWC  34
A.PT.1993.TER.GQ331137             CKRPGNKTVLPITLMSGLVFHSQP-INRRPRQAWC  34
A.PT.1993.JAU.GQ331133             CKRPGNKTVLPITLMSGLVFHSQP-LNTRPRQAWC  34
A.JP.2008.NMC786.AB499691          CKRPGNKTVVPITLMSGLVFHSQP-INRRPKQAWC  34
A.IN.1999.NARI_H2_10_2.DQ870459    CKRPGNKTVVPITLMSGLVFHSQP-INKRPKQAWC  34
A.GW.1989.89GW1816.L76424          CRRPGNKTVVPITLMSGLVFHSQP-INTRPRQAWC  34
A.GW.1989.89GW1812.L76422          CRRPGNKTVVPITLMSGLVFHSQP-INKRPRQAWC  34
A.GW.1989.89GW2300.L76428          CRRPGNKTVVPITLMSGLVFHSQP-INKRPRQAWC  34
A.FR.-.96325.AF170050              CKRPGNKTVVPITLMSGLVFHSQP-INKRPRQAWC  34
A.FR.-.96326.AF170038              CKRPGNKTVVPITLMSGLVFHSQP-INKRPRQAWC  34
A.IN.2000.NARI_H2_14_6.DQ870471    CKRPGNKTVVPITLMSGLVFHSQP-INKRPRQAWC  34
A.DE.-.BEN.M30502                  CKRPGNKTVLPITLMSGLVFHSQP-INTRPRQAWC  34
A.DE.-.BEN.NC_001722               CKRPGNKTVLPITLMSGLVFHSQP-INTRPRQAWC  34
A.GH.-.GH1.M30895                  CKRPGNKTVVPITLMSGLVFHSQP-INTRPRQAWC  34
A.PT.1992.93PTHDESC_21.JX219597    CKRPGNKTVTPITLMSGLVFHSQP-INTRPRQAWC  34
A.GW.1989.89GW1010.L76430          CKRPGNKTVVPITLMSGYRFHTQPVINTRPRQAWC  35
A.US.1988.E4420.U24388             CKRPGNKTVVPITLMSGMKFHSRPVINKKPRQAWC  35
A.IN.1995.CRIK_147.DQ307022        CKRPGNKTVTPITLMSGYKFHSRPVINTRPKQAWC  35
A.PT.1993.MIL.GQ331134             CKRPGNKTVVPITLMSGAKFHSRPVINTKPKQAWC  35
                                   ****** ** *******  **  * **  * ****
```

Figure S1. Alignment of the V3 loop of HIV-2 sequences obtained from Los Alamos database (http://www.hiv.lanl.gov, accessed on 18 February 2022). V3 sequences from (**a**) asymptomatic **(b)** symptomatic and **(c)** AIDS diagnosed individuals. Amino acids are denoted by single-letter code. The red boxes highlight the positions 18, 19, 25, 28 and 29 of the V3 loop (numbered according to the first Cys (C) residue), (-) represents a gap and * indicates conserved residues. Yellow highlight represents sequences with valine insertion in position 25.

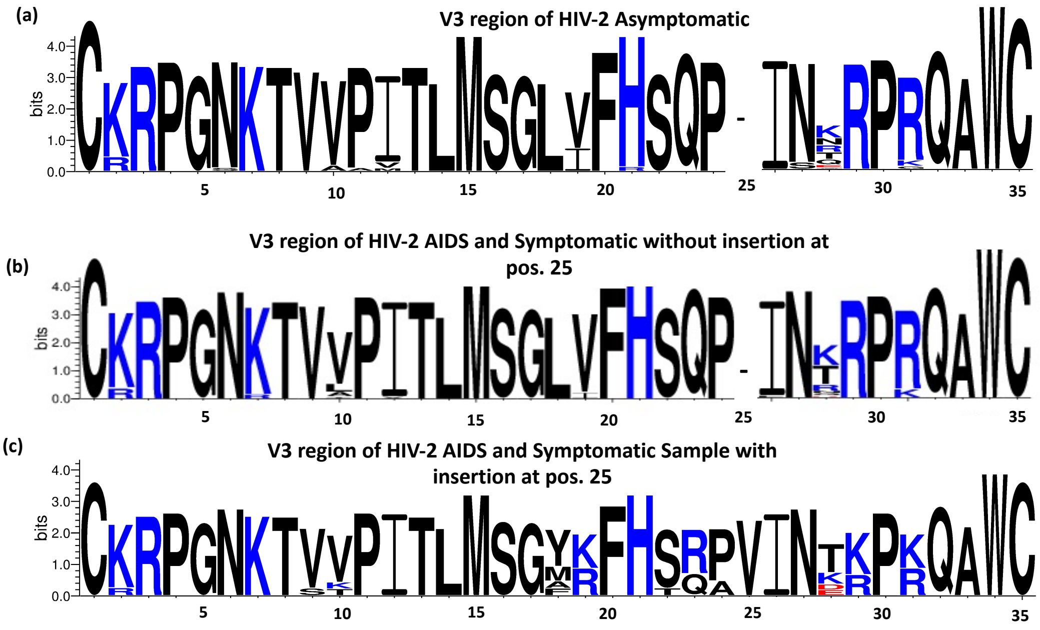

Figure S2. Genetic diversity of V3 loop of HIV-2 Env. HIV-2 Env V3 sequences from **(a)** asymptomatic, **(b)** symptomatic and **(c)** AIDS diagnosed individuals were collected from the Los Alamos National Laboratory (LANL) HIV sequence database (accessed on 18 February 2022)(http://www.hiv.lanl.gov). ClustalW was used to align multiple amino acid sequences, and variability of sequences was schematically visualized using Weblogo. Positively charged amino acids (H, K, R) highlighted in blue and negatively charged amino acids represented in red (D, E).

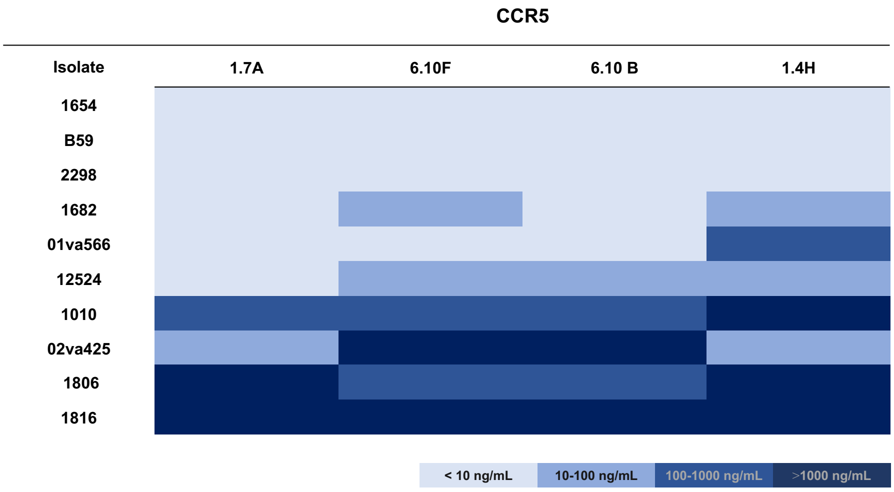

Figure S3. Neutralizing titers of mABs against HIV-2 primary isolates for infection of CCR5 expressing GHOST (3) cells.

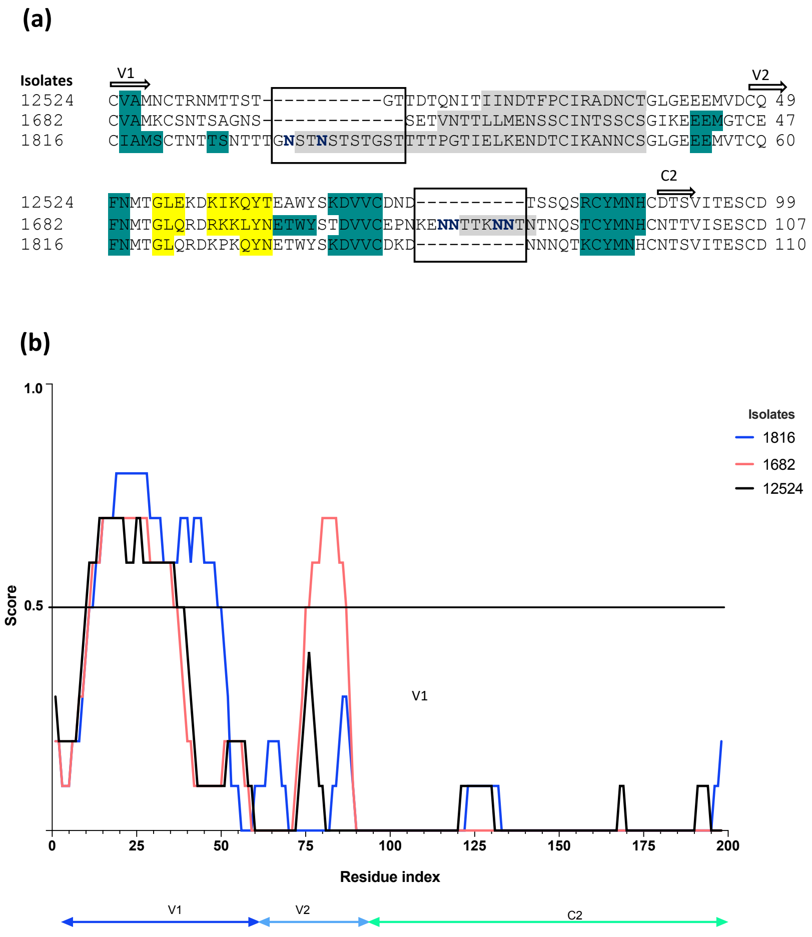

Figure S4. Comparison of V1/V2 region of 12524 R5/X4 user isolate. (**a**) Alignment of V1/V2 regions of HIV-2 1816, 1682 and 12524 isolates. Yellow highlight represents beta strand, green highlight represents alpha-helix, while the grey represents the predicted disordered regions. Amino acids are presented by single-letter code. Black boxes indicate regions with insertions. Potential N-linked glycosylation sites in the insertions are represented in dark blue. (**b**) Disorder prediction of the V1/V2 and C2 regions of HIV-2 12524 envelope. Prediction was performed by using IUPred3 and SPOT-Disorder2 online servers [30,31]. Cut-off is represented with black line at score 0,5.

Table S1. Sequences of HIV-2 V1 and V2 regions used in analysis for insertion in the variable region. Data regarding sequences including sequence name, status and "given name" in the alignment

Status	ID	ID in alignment
AIDS	A.DE.-.BEN.M30502	AIDS1
	A.DE.-.BEN.NC_001722	AIDS2
	A.GH.-.GH1.M30895	AIDS3
	A.IN.1995.CRIK_147.DQ307022	AIDS4
	A.IN.1999.NARI_H2_10_2.DQ870459	AIDS5
	A.IN.2000.NARI_H2_13_1.DQ870467	AIDS6
	A.IN.2000.NARI_H2_14_6.DQ870471	AIDS7
	A.IN.2001.NARI_H2_11_2.DQ870462	AIDS8
	A.PT.1992.93PTHDESC_21.JX219597	AIDS9
	A.PT.1993.JAU.GQ331133	AIDS10
	A.PT.1993.MIL.GQ331134	AIDS11
	A.PT.1993.TER.GQ331137	AIDS12
Asymptomic	A.GM.-.MCN13.AY509259	AYSM1
	A.GM.1990.CBL24.U05353	AYSM2
	A.IN.1995.NARI_H2_5_4.DQ870442	AYSM3
	A.IN.1996.NARI_H2_4_3.DQ870439	AYSM4
	A.IN.1999.NARI_H2_2_4.DQ870433	AYSM5
	A.IN.1999.NARI_H2_6_3.DQ870445	AYSM6
	A.IN.1999.NARI_H2_7_2.DQ870448	AYSM7
	A.IN.2000.NARI_H2_8_3.DQ870451	AYSM8
	A.IN.2000.NARI_H2_15_3.DQ870472	AYSM9
	A.IN.2000.NARI_H2_16_4.DQ870475	AYSM10
	A.IN.2001.NARI_H2_1_4.DQ870430	AYSM11
	A.IN.2001.NARI_H2_3_3.DQ870436	AYSM12
	A.PT.1997.MS.GQ331136	AYSM13
	A.PT.2000.00PTHDECT_20.GU983917	AYSM14
	A.SN.1986.ST_JSP4_27.M31113	AYSM15
	A.PT.1997.ARM.AJ001163	AYSM16
	A.PT.1997.ARM.L76739	AYSM17
Symptomatic	A.IN.-.NIM_1.DQ473391	SYM1
	A.IN.1999.NARI_H2_9_3.DQ870454	SYM2
	A.IN.1999.NARI_H2_12_3.DQ870463	SYM3
	A.IN.1999.NARI_H2_17_2.DQ870478	SYM4
	A.PT.-.ALI.AF082339	SYM5
	A.PT.1997.ETP.GQ331132	SYM6
	A.PT.1997.MLC.GQ331135	SYM7
	A.PT.2003.03PTHDECT_47.GU983933	SYM8
	A.PT.2003.03PTHDECT_50.GU983934	SYM9
	H2_01_AB.CI.1990.7312a.FB293476	SYM10
	H2_01_AB.CI.1990.7312A.L36874	SYM11

(a)

```
              V1
AYSM1    CVAMRCNNTDARNTTTPTTAS-------PRTIKPVTEISENSSCIRANNCS-GLGEEEVV
AYSM2    CVAMKCNNTATTRGGST------SHVHNCCTATGAMEINETSPCVSTNNCT-GLGDEEMI
AYSM3    CVAMNCSNITSSNTSAG-----------NTPTSTTLEIKENSSCISNNTCP-GLREEEIV
AYSM4    CVAMNCNTTTRNTTTHSNN--------TTTTTPSTLEINETSPCASANTCT-GLEEEEIA
AYSM5    CVAMSCNNTSTGNTTTS---------------T-NVSEVNDTSPCARANNCP-GLAEEEMV
AYSM6    CVAMNCSKNSGSTS-TPPPST--TSAHTIIPTPNVTEINENSSCIRTNNCS-GLGEEEIV
AYSM7    CVAMNCSSTGTGNTT---------------TTAATEPLIRENSSCIRTNNCS-GLGQEEIV
AYSM8    CVAMNCSRKNSGSTSTGNTTT--ASTSATTSTSEPMINESSSCIRTNNCS-GLREEEMV
AYSM9    CVAMNCSRDNTSTGNT--------------TTSTPTSIISENSSCIHENNCS-GLGDEDIV
AYSM10   CVTMKCNTSMATT-----------------TPKATETTINETFPCIRADNCA-GLGDEEIV
AYSM11   CVAMSCNNTSTGDTTTP---------------ASSSLEVNETSPCTHTDSCL-GLAEEEMV
AYSM12   CVAMNCNTTTRNTTTHSNN--------TTTTTPSTLEINETSPCASANTCT-GLEEEEIA
AYSM13   CVAMNCSSMGNTTATTST----------TTTTTDEKELDKNDTCIKGDNCT-GLGMEDMV
AYSM14   CVAMRCMI-----------------------------NESSSCIKTDNCT-GLEDEEMV
AYSM15   CVAMRCNSTTAKNTTS----------TPTTTTTANTTIGENSSCIRTDNCT-GLGEEEMV

SYM1     CVAMNCSRNDTSRSTTASSSTSTATSTTKSTTPETPEIRENSPCIRANNCS-GLGEEEIV
SYM2     CVTMKCNTGMATT-----------------TPKATETTINETFPCIRADNCA-GLGDEEMV
SYM3     CVAMNCSINSNNTRAE----------------NTSKIIITNKTGCINTNDSP-GLGKEEVV
SYM4     CVAMNCSRNITSTATPTI---------STNTSKTPEIEINETSPCVSTNSCP-GLGDEEIV
SYM5     CIAMKCSNISTESTTTSPS----------PGSTLKPLINESDPCIKADNCPRGLGDEEMV
SYM6     CVAMQCNNTKTGSTTTSPT------TSAATTPQIPKVVDDSSSCAGNDTCP-GLGDEETV
SYM7     CVAMKCTNMTTPRAATTT---------TPTTTTSNKTLIDENSPCIRTNNCS-GLGEEDMM
SYM8     CVAMTCIL-----------------------------NENSSCIKENNCT-GIGDEEMI
SYM9     CVAVTCIL-----------------------------NENSSGIKENNCT-GIGDEEMI
SYM10    CVAMSCNSTTATTTPPSTT----NNTTTTEPTTGGPEINETFPCMRTDNCT-GLGEEEMV
SYM11    CVAMSCNSTTATTTPPSTT----NNTTTTEPTTGGPEINETFPCMRTDNCT-GLGEEEMV

AIDS1    CVAMNCSRVQGNTTTPNPRTS--SSTTSRPPTSAASIINETSNCIENNTCA-GLGYEEMM
AIDS2    CVAMNCSRVQGNTTTPNPRTS--SSTTSRPPTSAASIINETSNCIENNTCA-GLGYEEMM
AIDS3    CVAMSCNSTTN---------------NTTTTGSTTGMSEINETSPS-YSDNCT-GLGKEEIV
AIDS4    CVTMRCNTSTTTTTTAPTSTS-----AGSTATPKPIMVNENTSCMHANNCS-GLGEEEVV
AIDS5    CVAMNCTPVPTNIST-PNI---------TATNTSKTLEINETSPCISANDCS-GLEEEEMV
AIDS6    CVAMNCSKKGNNTRTGNVTTT--AATTTTTTKNNITEINETSPCIRNNTCS-GLRDEEIV
AIDS7    CIAMSCRNTSTGNTTRARH------TPT-TTIKTPIEINESNPCIRGNNCS-GLGEEEMV
AIDS8    CVAMNCSRNSSNTSTGNTNATTSSTSAPATSPIPNLIINDSSTCINKSSCT-GLGREEIV
AIDS9    CVAMTCNSTRSNNTESN-----------TNTTAEAPEINDNSSCIRANNCS-GLGEEETV
AIDS10   CVAMNCTGNTTKSTTPKPSNT--PSVYTSSVSTTMKVLEENDPCIKESNCT-GLGREEMV
AIDS11   CVAMTCNYTDTSNATTGNSSTSIPTTTTTTTTQTAAEVGTGSPCIRKDSCP-GLGEEEVV
AIDS12   CVAMTCNKTENTTTATD---------------PIQTPEINDTSSCAHTDNCT-GLGEEEMV
```

(b)

```
                             V2 insertion                C2
                             ==>                         ==>
AYSM1        WYSKD-VVCEGNG----------TTDTCYMNHCNTSVITES
AYSM2        WYSKD-VVCDSTPGNS------TNQSRCYMNHCNTSVITES
AYSM3        WYSRD-VVRE-----NN----TNNTSRCYMNHCNTSVITES
AYSM4        WYSRD-VVCETKT--NN----TSNQSRCYMNHCNTSVITES
AYSM5        WYSSD-VVCEATGN-------TSNQSRCYMHHCNTSVIRVS
AYSM6        WYSSD-VVCDN------------NSSRCYMNHCNTSVITES
AYSM7        WYSSD-VVCEPVTNT--------TNQKCYMNHCNTSVITES
AYSM8        WYSSD-VVCETPSN-------APNQSRCYMSHCNTLVITES
AYSM9        WYSND-VVCEPIKADNT-SNNTSNRERCYMNHCNTSVIKES
AYSM10       WYSED-VVCE-----NS----TANSSKCYMNHCNTSVITES
AYSM11       WYSSD-VVCETTGNTSN----TSNQSRCYMNHCNTSVITES
AYSM12       WYSRD-VVCETKT--NN----TSNQSRCYMNHCNTSVITES
AYSM13       WYSRD-VVCENSSDSKT-ANKSKDNMRCYMNHCNTSVITES
AYSM14       WYSRD-VVCENSSDSKT-ANKSKDNMRCYMNHCNTSVITES
AYSM15       WYSTD-VVCDTSSGN-T-SE--ENTTRCYMNHCNTSVITES
AYSM16       WYSKD-VVCD----------------RCYMNHCDTSVITES
AYSM17       WYSKD-VVCESNDTK--------KEKTCYMNHCNTSVITES

SYM1         WYSSD-VVCEANAT--------LNQSRCYMKHCNTSVITES
SYM2         WYSSD-VVCGANETDNS-SQ--TSYSRCYMNHCNTSVITES
SYM3         WYSKD-VVCETIGNTSA---ENTTARRCYMNHCNTSVITES
SYM4         WYSKD-VVCEEATNTSG----TTNTSRCYMNHCNASVITES
SYM5         WYSKD-VVCEPFNTT-------TNQTRCYMNHCNTSVITES
SYM6         WYSKD-VVCAKTNTANG----TKADSTCYMSHCNTSVIKES
SYM7         WYSKD-VVCDKA-DNS------ANQTRCYMNHCNTSVITES
SYM8         WYSKDRVVCDK----------------CYMNHCNTSVIRES
SYM9         WYSKD-VVCDK----------------CYMNHCNTSVIRES
SYM10        WYSKD-VVCESNN-AS------DGRDRCYMNHCNTSVITES
SYM11        WYSKD-VVCESNN-AS------DGRDRCYMNHCNTSVITES

AIDS1        WYLED-VVCDNTT-----------AGTCYMRHCNTSIIKES
AIDS2        WYLED-VVCDNTT-----------AGTCYMRHCNTSIIKES
AIDS3        WYSKD-VVCESNN-TK------DGKNRCYMNHCNTSVITES
AIDS4        WYSRD-VDCEPDSTT--------NSRKCYMNHCNTSVITES
AIDS5        WYSSD-VVCNE------------NQTRCYMNHCNTSVITES
AIDS6        WYSSD-VVCEE------------NQTRCYMNHCNTSVITES
AIDS7        WYSSD-VDCEATNT-------SSGQSRCYMKHCNTSVIRES
AIDS8        WYSKD-VTCEANRNG-------TDRIRCYMSHCNTSVITES
AIDS9        WYSRD-VVCENSSDSETTGNSSRNNMRCYMNHCNTSVITES
AIDS10       WYSRD-VVCESYNETNE-NN--ESQSRCYMNHCNTSVITES
AIDS11       WYSKD-VVCERNVTA--------KKDRCYMNHCNTTVITES
AIDS12       WYSKD-VICKTKN----------NTTRCYMNHCNTSVITES
```

Figure S5. Alignment of V1/V2 region of HIV-2 sequences. From **(a)** Alignment of V1 region from asymptomatic individuals, symptomic and AIDS patients. **(b)** Alignment of V2 region from asymptomatic, symptomic and AIDS patients. Amino acids are denoted by single-letter code. The red boxes highlight the insertions in the V1 and V2 regions.

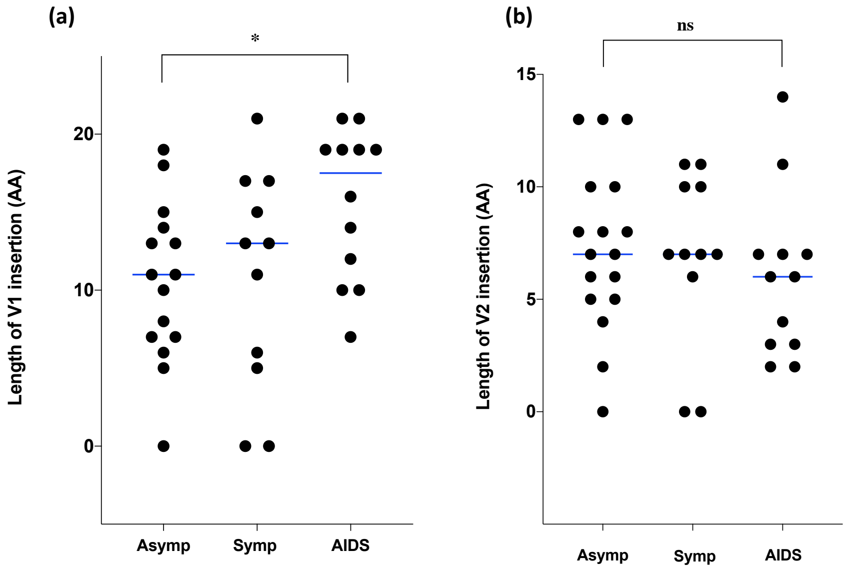

Figure S6. Length of insertions in the V1 and V2 region between asymptomatic, symptomic and AIDS groups. (a) Length of V1's insertion was significantly different between asymptomatic and AIDS groups. Statistical differences calculated according to non-parametric Mann-Whitney analysis, * P = 0,0162). **(b)** Comparison of length of V2's insertion did not differ any of the groups.

Table S2. Primers used for sequencing of HIV-2 *env*. HIV-2 BEN was used as a reference strain (GenBank accession number M30502), (F: Forward; R: Reverse).

Primer	Sequences	Position according to HIV-2 BEN *env*
F31 (F)	5' TGT TTy TTr mAm Aar GGr CTC G 3′	6040-6061
F41 (F)	5' AAT TTG TrA CTG TyT TCT ATG G 3′	6212-6229
F42 (F)	5′ ACT GTy TTC TAT GGm rTr CCC 3′	6216-6239
F43 (F)	5' TTC TAT GGm rTr CCC GCG TGG 3′	6222-6242
KH2OF (F)	5' GAG ACA TCA ATA AAA CCA TGT GTC 3′	6420-6443
CRSEQ-3 (F)	5' CAT TGC AA CAA TCr GTC ATy A 3′	6756-6777
CRSEQ2_F (F)	5′ Gay AAG CAC TAT TGG GAT GC 3′	6789-6808
CRSEQ5 (F)	5′ ATG TGG ACT AAC TGC AGr GGA 3′	7308-7328
TH2OR (R)	5' TTC TGCCAC CTC TGC ACT AAA GG 3′	7549-7571
CRSEQ7+AGT (F)	5′ AGT GCA GCA ACA GCA ACA GC 3′	7787-7806
CRSEQ-8 (R)	5' GAG AAA ACA GGC CTA TAG CC 3′	8262-8281
CRSEQ9+G (F)	5′ GGC TAT AGG CCT GTT TTC TC 3′	8262-8281
R3 (R)	5' ATC TAy ATC ATC CAT ATT yTG yTG 3′	8806-8829
R2 (R)	5′ TGT TTy TTr mAm Aar GGr CTC G 3′	8815-8839
